# Cutoff value for predicting success in triathlon mixed team relay

**DOI:** 10.3389/fspor.2023.1096272

**Published:** 2023-04-17

**Authors:** T. Ledanois, I. Hamri, Q. De Larochelambert, S. Libicz, J. F. Toussaint, A. Sedeaud

**Affiliations:** ^1^ URP 7329—IRMES, Université Paris Cité, INSEP, Paris, France; ^2^ FFTri, Fédération Française de Triathlon, Saint-Denis, France; ^3^ Centre D'Investigation en Médecine du Sport, Hôtel-Dieu, Assistance Publique—Hôpitaux de Paris, Paris, France

**Keywords:** endurance, triathlon, performance, mixed relay, practical application

## Abstract

**Introduction:**

The Mixed-Team-Relay (MTR) triathlon is an original race format present on the international scene since 2009, which became an Olympic event at the Tokyo 2020 Games. The aim of this study was to define the probabilities of reaching a victory, a podium, or a finalist rank in a relay triathlon, according to the position of any of the four relayers (Women/Men/Women/Men) during each of the four segments (leg) of the race.

**Methods:**

All MTR results from the World Series, Continental Championships, World Championships from 2009 to 2021 and Tokyo 2020 Olympics have been collected. We calculated the set of probability frequencies of reaching a given final state, according to any transient state during the race. All results are compared with a *V*' Cramer method.

**Results:**

The frequency of winning is similar at the end of Leg 1 for TOP1 (first position) and TOP2-3 (second and third positions). Then, a difference in the winning-associated frequencies is first observed after the Bike stage of Leg 2, where 47% of TOP1 athletes will win, *vs* 13% of the TOP2-3.

**Discussion:**

This difference continually increases until the end of the race. Legs 2 and 3 are preponderant on the outcome of the race, the position obtained by each triathlete, especially in swimming and cycling, greatly influences the final performance of the team. Leg 1 allows to maintain contact with the head of the race, while Leg 4 sets in stone the position obtained by the rest of the team.

## 1. Introduction

Triathlon mixed team relay (MTR) is the newest format of triathlon, which consists of a consecutive sequence of swimming, biking, running, and two transition phases on a supersprint format (250–300 m swimming, 5–8 km cycling, and 1.5–2 km running) (1), which is carried out consecutively by the four relay triathletes (women/men/women/men). World Triathlon has defined this as being the relay order until 2021. The MTR format is present in the calendar of the International Federation with a World Championship since 2009 and is included for the first time in the program of the Summer Olympic Games of Tokyo 2020. Furthermore, the recent increase in the number of MTR races in the World Triathlon Championship Series (WTCS) has offered more possibilities to analyze more races. The stakes of this format are important as they allow athletes who qualify for the Olympic Games on the relay to also participate in the individual race. Nations can strategically concentrate on having strong relay teams in the Olympic qualification period, as qualifying a team would automatically send their four athletes to the individual race (1).

Several studies have highlighted optimal strategies to reduce the energy cost during the race and increase the winning probabilities, with a correlation between exiting the water in the first positions and victory, in Olympic distance (2, 3). Cycling is likely to be influenced by drafting, as it relates to the difference in energy cost of riding in a peloton vs. riding alone (4). Drafting also reduces the energy expenditure for the rest of the event (5). In addition, maintaining one's position in the first pack is a determining factor in the final performance (6). The running leg strong affects final performance, where we observe a maximal effort of the athletes in the initial and final phases, a pattern called “Parabolic pacing” (7, 8). However, it was found that the influence of each split depends on the length of the triathlon and that the shorter the distance, the more swimming correlates with the outcome of the race (9). These authors highlight that triathletes in leading positions going into the run could have a psychological advantage over the chasing athletes, allowing them to perform better (3, 9). To date, only a few articles (10, 11) have studied the MTR race format. They have mainly focused on the physiological requirements of this format, bringing practical applications to help coaches plan and execute race tactics or develop training strategies. The frequent high-intensity bursts observed in all three disciplines and the positive pacing strategies are well-developed anaerobic qualities in elite triathletes (10). Recently, authors have shown how specific the skills of each relayer need to be (11); in particular, triathletes skilled in racing in a group seem like a better choice for Legs 1 and 2, while those who are good at pacing themselves and skilled in racing in nondrafting situations seem to be better suited for Legs 3 and 4.

The aim of this study was to define the probabilities of reaching a victory, podium, or finalist rank in a relay triathlon according to the position during each segment of the race. This study was designed to bring practical suggestions to the coaches and technical staff to form a successful MTR team by composing each leg to weigh on the race dynamics.

## 2. Methods

### 2.1. Data collection

This analysis included data from elite national teams competing from 2009 to 2021. All performances in World Triathlon races were collected: WTCS, World Championship (WChamp), Continental Championship (ConChamp), and Olympic Games (OG) on the MTR. Within 38 MTR races, 394 different teams have finished the race, representing 1,576 performances realized by 2,924 male and female athletes. The ITU race data are publicly available and extracted from the World Triathlon database (www.triathlon.org). All the data were downloaded using a script on R querying by a public API (12).

### 2.2. Data analysis

All race times were collected (swim, bike, run, and transition) to track the positions of each triathlete during the relays. Nonfinishers (DNF, DSQ) were considered to finish last, classified in the order of the teams' withdrawal from the race. Each racer is an iteration of a supersprint triathlon; they were coded as Legrelayer number—sport. The transition phases were excluded from the analysis because the position changes are an information redundancy with the previous phase. Indeed, in our dataset, all the transitions did not bring any change to the ranking between the entry and exit of the transition phase. The relay teams were divided into four groups according to their positions throughout the race (state), the first (TOP1), second and third (TOP2–3), fourth to eighth (TOP4–8) positions, and teams above the eighth place (overTOP8). The fourth to eighth (TOP4–8) positions express the possible transitional state to the finalist position. It represents the team's potential medalist; considering them allows us to visualize whether they have a probability of reaching the podium. The final states observed were the “Winner” (1st rank), “Medalist” (1st to 3rd rank), and “Finalist” (1st to 8th rank).

### 2.3. Statistical analysis

The frequency is expressed with a 95%confidence interval. This represents the number of teams that reached the final state in this transient state. A Student’s *t*-test was used to compare the time, ranking, and time difference at the head of each leg, and the results are expressed as means and standard deviations. A chi-square test of adequacy was performed at each section of the race to test Hypotheses *H0*: “rank has no influence on final ranking” and H1: “position is determinant of final ranking.” In addition, the result is supplemented with an effect size value *Cramer’s V* (13–15) to express the magnitude of the probability of reaching the final states. The level of statistical significance was set at *p* ≤* *0.05, and the *V* values were considered as small ≤ 0.06, medium ≤ 0.17, large ≤ 0.29, and very large effect > 0.29. The chi-square test of adequacy does not meet all the conditions of application, in particular, because the theoretical number of participants is fewer than 5. We will express this difference between the groups with only the effect size and 95% CI. All the data were processed and analyzed using R v.1.4.1106.

## 3. Results

### 3.1. Position and time at the head of the race

The average differential times (difference in time from the time of the first triathlete in that segment) for each classification category and each race component are presented in Table 1. For example, the 1st relay runners (Leg 1) of the winning teams in the race recorded a significantly faster time (333.45 ± 45.24) than the first runner of the Finalist teams (347.59 ± 53.77) and the Finisher teams (366.27 ± 98.64) (Table 1). This is verified by the average rankings, which follow the same trend (Table 1). For example, in Leg 2, athletes competing in the Winner teams already show lower ranks in swim, bike, and run (3.08 ± 2.64; 2.58 ± 2.11; 2.21 ± 1.68) than their Finalist (6.46 ± 2.96; 6.3 ± 2.73; 6.21 ± 2.59) and Finisher (13.04 ± 4.91; 13.24 ± 4.72; 13.34 ± 4.6) counterparts, respectively (Table 1). The position in the rankings during the race is significantly different between the Winners and Medalists vs. Finalists vs. Finishers. The difference in rank during the race becomes significant between Winners and Medalists at the third relay, and this will be observed in the rest of the race (Table 1). If we only observe the raw times, it should be noted that the times on each segment do not differ significantly between the Winner, Medalist, and Finalist groups but each of these three groups has a significantly different time from the Finisher group on each segment of the relay (Table 1).

**Table 1:** Average ranks, times, and differential times in each component of the relay for the winner, medalist, finalist, and finisher states (and standard deviation).

		Leg 1			Leg 2			Leg 3			Leg 4		
		Swim	Bike	Run	Swim	Bike	Run	Swim	Bike	Run	Swim	Bike	Run
Winner	Rank	5.55 ± 4.33 (c,d)	3.45 ± 3.22 (c,d)	3.18 ± 2.86 (c,d)	3.08 ± 2.64 (c,d)	2.58 ± 2.11 (c,d)	2.21 ± 1.68 (b,c,d)	2.16 ± 1.55 (b,c,d)	1.74 ± 1.37 (b,c,d)	1.47 ± 0.98 (b,c,d)	1.39 ± 0.68 (b,c,d)	1.26 ± 0.5 (b,c,d)	1 ± 0 (b,c,d)
	Time	240 ± 38.86 (d)	625 ± 70.54	333.45 ± 45.24 (d)	225.09 ± 25.47 (d)	574.52 ± 66.49 (d)	296.55 ± 37.32 (d)	247.82 ± 26.94 (d)	629.18 ± 74.27 (d)	334.58 ± 40.6 (c,d)	227.73 ± 23.92 (d)	582.39 ± 64.95 (d)	300.7 ± 46.24
	Diff. 1st.	18.12 ± 54.42 (d)	7.55 ± 11.81 (c,d)	12.94 ± 15.75 (c,d)	10.64 ± 10.57 (c,d)	7.45 ± 11.53 (b,c,d)	8.73 ± 11.97 (b,c,d)	6.64 ± 9.47 (b,c,d)	3.85 ± 8.97 (b,c,d)	4.79 ± 9.25 (b,c,d)	3.48 ± 7.01 (b,c,d)	0.64 ± 1.27 (b,c,d)	0 ± 0 (b,c,d)
Medalist	Rank	5.03 ± 3.72 (c,d)	3.7 ± 2.64 (c,d)	3.28 ± 2.02 (c,d)	3.38 ± 2.41 (c,d)	3.21 ± 1.95 (c,d)	3.2 ± 1.95 (a,c,d)	2.8 ± 1.78 (a,c,d)	2.93 ± 1.59 (a,c,d)	2.67 ± 1.05 (a,c,d)	2.63 ± 1.08 (a,c,d)	2.54 ± 0.96 (a,c,d)	2.5 ± 0.5 (a,c,d)
	Time	238.57 ± 40.07 (d)	628.26 ± 68.78	334.76 ± 43.91 (d)	224.53 ± 25.72 (d)	579.39 ± 63.58 (d)	300.35 ± 39.4 (d)	248.42 ± 28.21 (d)	635.73 ± 71.66 (d)	341.24 ± 49.01 (d)	227.26 ± 24.23 (d)	589.61 ± 71.02 (d)	302.52 ± 44.47
	Diff. 1st.	16.69 ± 53.93 (d)	9.58 ± 13.23 (c,d)	17 ± 18.94 (c,d)	14.14 ± 17.96 (c,d)	15.53 ± 24.29 (a,c,d)	20.59 ± 25.97 (a,c,d)	19.11 ± 27 (a,c,d)	23.05 ± 32.78 (a,c,d)	31.41 ± 36.75 (a,c,d)	29.64 ± 37.18 (a,c,d)	33.94 ± 46.18 (a,c,d)	36.14 ± 37.19 (a,c,d)
Finalist	Rank	7.55 ± 4.4 (a,b,d)	6.85 ± 3.41 (a,b,d)	6.75 ± 3.23 (a,b,d)	6.46 ± 2.96 (a,b,d)	6.3 ± 2.73 (a,b,d)	6.21 ± 2.59 (a,b,d)	6.27 ± 2.42 (a,b,d)	6.08 ± 2.29 (a,b,d)	6.16 ± 2.13 (a,b,d)	6.14 ± 2.04 (a,b,d)	6.04 ± 1.69 (a,b,d)	6 ± 1.42 (a,b,d)
	Time	243.87 ± 41.66 (d)	637.79 ± 73.88	347.59 ± 53.77 (d)	226.48 ± 25.62 (d)	587.06 ± 65.96 (d)	307.08 ± 42.21	256.78 ± 32.7 (d)	654.13 ± 79.13 (d)	353.68 ± 53.22 (a,d)	232.36 ± 27.09 (d)	597.73 ± 65.84 (d)	311.49 ± 46.86
	Diff. 1st.	21.99 ± 55.04 (d)	24.91 ± 31.58 (a,b,d)	45.36 ± 50.74 (a,b,d)	44.44 ± 53.26 (a,b,d)	53.64 ± 65.62 (a,b,d)	66.14 ± 74.3 (a,b,d)	73.01 ± 83.05 (a,b,d)	96.09 ± 103.79 (a,b,d)	118.47 ± 123.06 (a,b,d)	121.8 ± 130.65 (a,b,d)	134.85 ± 144.65 (a,b,d)	146.48 ± 147.83 (a,b,d)
Finisher	Rank	11.66 ± 5.74 (a,b,c)	12.67 ± 5.19 (a,b,c)	12.87 ± 5.04 (a,b,c)	13.04 ± 4.91 (a,b,c)	13.24 ± 4.72 (a,b,c)	13.34 ± 4.6 (a,b,c)	13.4 ± 4.5 (a,b,c)	13.54 ± 4.31 (a,b,c)	13.59 ± 4.25 (a,b,c)	13.62 ± 4.2 (a,b,c)	13.72 ± 4.09 (a,b,c)	13.79 ± 4 (a,b,c)
	Time	258.53 ± 51.45 (a,b,c)	646.52 ± 83.43 ICI pas abc	366.27 ± 98.64 (a,b,c)	237 ± 26.37 (a,b,c)	602.07 ± 77.95 (a,b,c)	319.62 ± 92.99 (a,b)	266.45 ± 34.55 (a,b,c)	670.8 ± 85.27 (a,b,c)	359.2 ± 51.27 (a,b)	244.09 ± 32.47 (a,b,c)	610.25 ± 79.31 (a,b,c)	311.92 ± 46
	Diff. 1st.	40.07 ± 83.33 (a,b,c)	50.71 ± 49.5 (a,b,c)	94.96 ± 127.52 (a,b,c)	101.98 ± 123.33 (a,b,c)	135.13 ± 144.48 (a,b,c)	170.46 ± 249.62 (a,b,c)	184.58 ± 248.06 (a,b,c)	226.34 ± 239.79 (a,b,c)	250.73 ± 188.64 (a,b,c)	260.77 ± 198.51 (a,b,c)	287.31 ± 233.84 (a,b,c)	304.49 ± 237.67 (a,b,c)

Differential time is defined as the difference between an individual's split time for a component of the race and the head of the race for that component in the same race. Significance *t*-test were performed for the three variables. Letters a–d indicate statistically significant differences (*p*-value < 0.05): (a) Winner, (b) medalist, (c) finalist, and (d) finisher.

### 3.2. Positions and frequencies for winners

**Figure 1:**
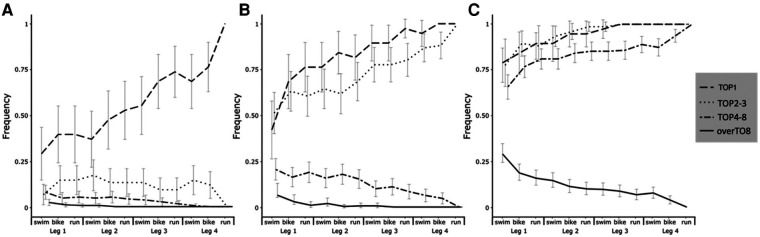
Frequency of reaching a Winner (**A**), Medalist (**B**), or Finalist (**C**) state depending on the state obtained during the MTR.

**Figure 2:**
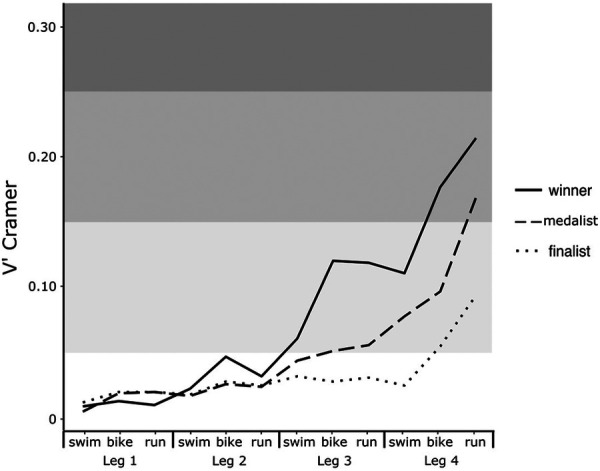
Evolution of the magnitude of *V*' Cramer calculated for each final state.

The frequencies of winning at the end of the race converge between TOP1 and TOP2–3 until Leg 2—bike. After this step, the frequencies differ between the two groups (Figure 1A). In addition, the 95% CI does not merge anymore. TOP4–8 and overTOP8 have a decreasing trend from Leg 1—swim with, respectively, 8% and 6% probability of finishing 1st (Figure 1A). Furthermore, the effect size shows a high increase in the difference in probabilities of achieving victory with the first time at Leg 2—bike (*V* > 0.06) and a second strong increase after Leg 3—bike (*V* > 0.13) (Figure 2).

### 3.3. Positions and frequencies for medalists

The observed frequencies of reaching the podium for TOP1 and TOP2–3 on each segment of the race are similar with an overlapping 95% CI except for Leg 2—bike, Leg 3—run, and Leg 4—bike (Figure 1B). The TOP2–3 and TOP4–8 transient states differ, with respective increases and decreases in the likelihood of reaching the podium at the end of the race after the Leg 1—bike segment (Figure 1B). On the other hand, the Cramer’s *V* method reveals a trend that increases the difference in the frequency of reaching the podium according to the group to which one belongs, notably with a strong increase after Leg 1—bike. The frequencies of winning at the end of the race converge between TOP1 and TOP3 until Leg 2—bike. After this step, the frequencies differ between the two groups (Figure 1A).

The probabilities of achieving the final state “Finisher” are the same for groups TOP1, TOP2–3, and TOP4–8 and strongly different for the overTOP8 group (Figure 1C). Furthermore, the teams in transient states overTOP8 have a declining observed frequency of reaching the final state “Winner,” “Medalist,” and “Finalist” as the race goes on (Figures 1A–C).

## 4. Discussion

The objective of this study was to analyze the contribution of each segment of the mixed relay triathlon to overall performance since the introduction of this format to the international calendar. The main idea was to find out whether there was a major moment in the outcome of the race along the relay.

### 4.1. Dynamic of races

The differences between the Winner, Medalist, Finalist, or Finisher teams are apparent throughout the race. Considering only the raw times achieved in each segment, no differences between the Winner, Medalist, and Finalist teams could be observed. Only the Finisher group differs from their better counterparts in rank and time since the beginning of the race. It can be noticed that the Winner and Medalist teams place themselves more in front than the Finalist teams from the first segment, with rapid differences. This strategy would aim not to create a chronometric difference but to place themselves near the head of the race, just like what was observed on the swimming course in the standard format, where swimming is a precursor for placing triathletes (16). In a second time, the difference in rank is created between the head of the race and the finalist and finisher on the second bike segment (Leg 2—bike). Then, once this difference is generated, there is real security by increasing and stabilizing the gap (Table 1). Indeed, it is only in Leg 3 that the average rank of the Winners differs from the other competitors, KG. Thompson refers to this phenomenon of temporization when the gap is created with the chasers on the individual triathlon, in particular, for conserving energy (17). The key information is that the first two triathletes must be able to move within the group and that once the gap is made, the third runner takes the lead on a solitary effort.

### 4.2. Legs 2 and 3 as tipping points

A major finding of this study is the observed strong increase in the frequency of victory for the team of the leading athlete on Leg 2—bike and on Leg 3—bike. These two parts of the race are thus crucial to focus on in team strategies to reach final victory (Figure 1A), notably because they are the segments with the most time spent racing and therefore exposure to create a gap (10). Moreover, the probability of winning with an athlete in the lead in these games increases after these two segments, respectively, with a strength of association expressed by Cramer's *V* that increases (Figure 2). These results provide practical applications to compose an MTR team according to the objectives defined beforehand by analyzing the results of all the major competitions of the last Olympic period.

### 4.3. Positions and frequencies for medalists

Moreover, we can notice a difference in the frequency of reaching the podium between TOP2–3 and TOP4–8 from Leg 1 (Figure 1B). This completes the conclusion of Quagliarotti (11), who claims that relays 3 and 4 are the most predictive of the final result, and for this reason, the frequency of winning and reaching the podium is strongly in favor of the TOP1 and TOP2–3 positions after Leg 2 (Figure 2). It is important to mention that teams positioned beyond the ninth place as soon as Leg 1 show a low frequency of coming back and a small probability of winning the race, which decreases all along the race (Figure 1C). We can see that it is important to maintain a position at the front of the race (TOP1—TOP2–3) and that a poor first leg is a major hindrance to victory and reaching the podium. This makes it an elimination race “from the rear.” This increase in adequacy after Leg 2 is mainly due to the formation of small packs or even the isolation of individual triathletes, which leads to the removal of the drafting effect, i.e., making athletes perform a time trial-type effort, that increases the energy cost to bridge the gap created by teammates in previous legs (18–20). The strategic characteristics of each relay bring into play physiological and psychological demands that are specific to MTR and to each leg of the relay, in addition to the particular demands of the shorter duration of the supersprint format (11). This is particularly true for the first two runners for whom the effort is close to one of an individual supersprint paired with the promiscuity of the other athletes, as well as the repetitions of high-intensity bursts (18, 21).

### 4.4. Consequences for relayers profiles

This leads us to believe that it is necessary to put triathletes who have the most technical skills in these two first legs (11) or executue precise movements with strong space–time constraints, such as for the transitions of a sprint or a standard distance (20). In contrast, the efforts of relayers 3 and 4 have the aim of grounding and securing the position given to them by their teammates. The adequacy test, which highlights the differences of probability to reaching the podium for TOP4–8 vs. TOP1–TOP2–3 (Figure 2), suggests that as the gaps are already created and as triathletes become isolated from one another, the effort of the 3rd and 4th relayers would be smoother in intensity (11) and would be closer to an individual time trial effort. The results of this study suggest that the MTR is not an addition of the performances of four supersprints but a concordance of the composition of the relay according to the capacity of each relayer to correspond to the dynamics of their respective start order. This means that it is important to have a good idea of the skills of each relayer, in particular, their power output on the bike, their technical ability, and their capacity to open gaps, all elements that, paired with the right leg of the race, will lead to a victory or a podium. However, in terms of regulations, the composition of the relay teams is linked to the start list of the individual format of the Olympic event (1). This brings into play strategic choices on the objectives of each nation for the individual and team race, at the risk of not having a profile of athletes that do not meet a format. Regarding the composition of the relay teams, Leg 2 and 3 athletes must have a strong swimming ability to keep the team in contact with the race leader, considering the average positions of the “Winner “and “Medalist” (Table 1). This is confirmed by the ability to create a gap at this moment (Table 1) and by increasing the magnitude of the Cramer's *V* on the Leg 2—bike and Leg 3—bike. For these positions, it would be required to have specific “swimming—riding” skills.

### 4.5. Limitations

One of the limitations of this study is the small number of events over an Olympiad, which explains the use of the effect size to have a quantitative index from transient states to final states.

Finally, it was noted that this study takes into account the results of the MTR of the 2021 Olympiad, while the international federation made a choice to change the starting order for 2022/2024 (1) (men/women/men/women). This does not undermine the previous conclusions but could change the race dynamics by delaying the creation of gaps between teams.

Moreover, the set of races taken does not take into account the density, which, despite the constant level, can be variable from one race to another.

## 5. Conclusion

The introduction of the triathlon mixed team relay to the Olympic program has revealed a significant interest in increasing knowledge of this format. The composition and order of the relay team are the key to achieving the highest level of performance. It seems that the dynamic is that of a race by elimination race, i.e., by starting the bike with a large enough lead group on Leg 1, which will take advantage of the collaboration effects, while trying to shed opponents from the back. The dynamics then favor athletes that distance themselves off the front from the others on Legs 2 and 3, with the stronger cyclists creating an advantage for their team. Ultimately, to make the difference between being a candidate for the podium or soloing at the head of the race, the fourth triathlete must be able to race without the other athletes' draft, in particular, for swimming and cycling, to secure the best result possible for the team. The strategy is to work with the specific skills of triathletes or to get them to develop these competencies in training for them to specialize in a specific leg. This reveals that it is not just four supersprints in a row but a complementary profile combination.

## 6. Practical applications

According to these results, an MTR team composition must follow these recommendations, which are associated with the specific skills of relayers:

• Legs 1 and 2 athletes must ensure they stay as close as possible to the front of the race.
• Legs 3 and 4 athletes must be able to produce a solo time-trial-type effort to maintain the lead given to them.
• Legs 2 and 3 athletes must have a strong swim and bike combination, which is decisive for the final result.

This study reveals that MTR is not just four supersprints in a row but a complementary profile combination. MTR composition has to be precisely defined by the coaches to know as many MTR compositions as possible with their global workforce before a major event and to be able to evaluate all MTR compositions and strategies.

## Data Availability

Publicly available datasets were analyzed in this study. These data can be found here: https://www.triathlon.org/results.
